# Supplementary material for: Immunosuppressive Therapy Modifies Anti-Spike IgG Subclasses Distribution After Four Doses of mRNA Vaccination in a Cohort of Kidney Transplant Recipients
Source: Vaccines (Basel). 2025 Jan 25;13(2):123. doi: 10.3390/vaccines13020123 (PMC11860609; doi:10.3390/vaccines13020123)
Supplement: Supplementary file 1 [file vaccines-13-00123-s001.zip › vaccines-3403321-supplementary.pdf]

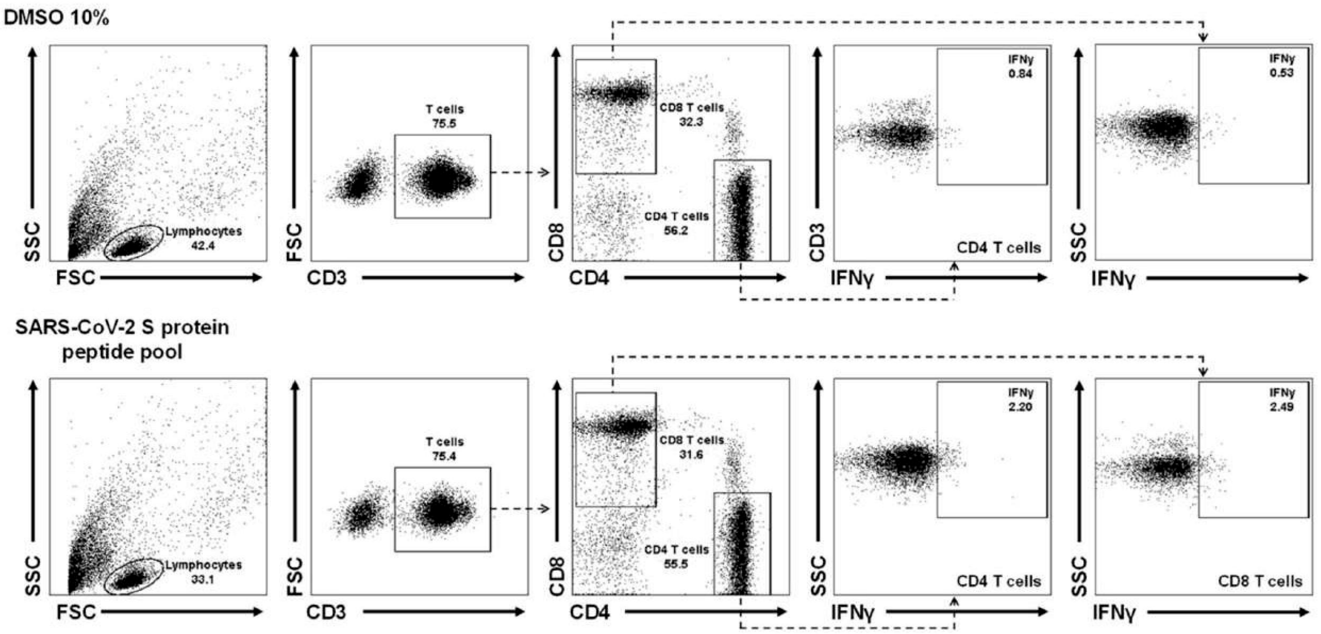

Supplementary Figure S1. Dot plot of negative control (DMSO 10%) and SARS-CoV-2 peptide pool to determine cellular response frequency in PBMC from KTR and DP.

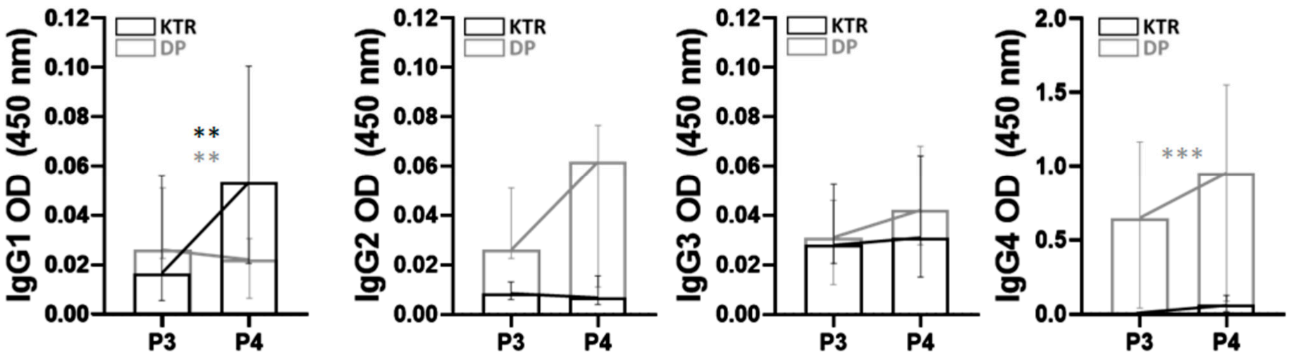

Supplementary Figure S2. Graphical representation of IgG subclasses kinetics in KTR an DP donors. P3: At 2 months after 3th dose. P4: At 2 months after 4th dose. KTR: Kidney Transplant recipients (n=60). DP: Dialysis Patients (n=12). IgG1 in KTR at P3 vs P4, p=0.010; IgG1 in DP at P3 vs P4, p=0.005; IgG4 in KTR at P3 vs P4 p=0.001. The cut-offs were established as follows: IgG1:0.0085; IgG2:0.0075; IgG3:0.0559; IgG4:0.018.

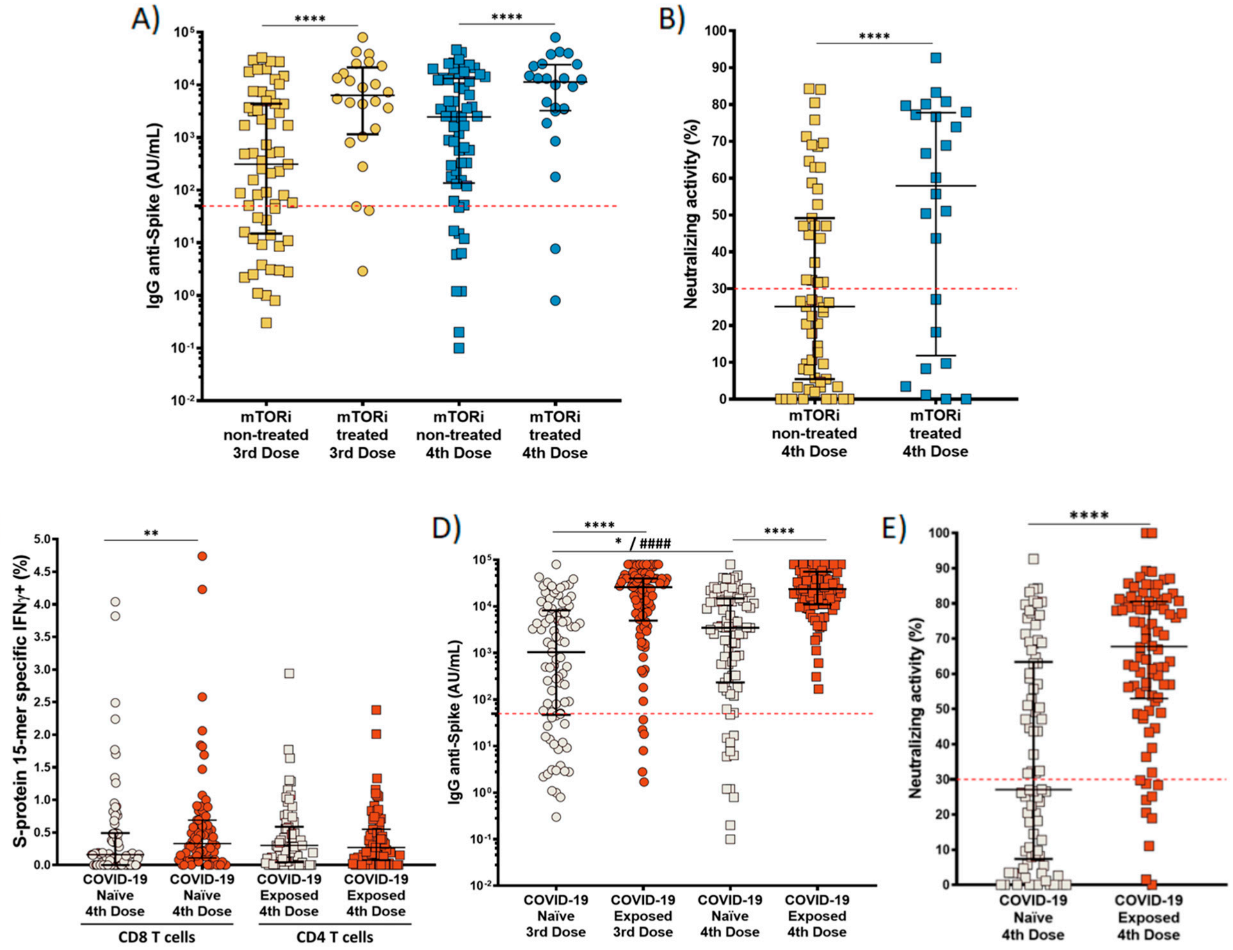

Supplementary Figure S3. IgG kinetics and neutralizing activity in KTR. A) mTORi treatment increases IgG response and neutralizing activity in KTR. mTORi showed association with SARS-CoV-2 IgG >50 AU/ml (technique positivity range) [OR 7.31 95% CI (0.92-58.99), p=0.056,] and neutralizing activity >30% (50th percentile of the sample) [OR 3.23 95% CI (1.17-8.90), p=0.030]. No association with cellular response was found. B) Serum neutralizing activity is increased in KTR treated with mTORi after 4 doses. C) KTR COVID-19 naïve developed higher CD8 T cell responses, measured by flow cytometry after 15-mer Spike peptides stimulation. D) IgG anti-Spike response in COVID-19 naïve and COVID-19 exposed individuals after 3 and 4 doses (23867 (12025-59346) vs 3640 (279-15940), p<0.0001) of mRNA-1237 vaccination. SARS-CoV-2 infection increased the IgG anti-Spike levels in serum of KTR. E) Serum neutralizing activity is increased by SARS-CoV-2 infection (74.4 (51.3-82.4) vs 30.0 (7.9-62.9), p<0.0001) in kidney transplant recipients. Unpaired M-W test, *: p<0.05, **: p<0.01, ****: p<0.0001; paired M-W test ####: p<0.0001. Data is expressed in AU/mL in order to show the original data obtained from the kit and the technique positivity. BAU/mL data is shown in the manuscript (BAU/mL = AU/mL × 0.142).
